# Ingestion rate estimated from food concentration and predatory role of copepod nauplii in the microbial food web of temperate embayment waters

**DOI:** 10.1093/plankt/fbad002

**Published:** 2023-02-01

**Authors:** Youta Sugai, Noriaki Natori, Kenji Tsuchiya, Megumi Nakagawa, Makio C Honda, Shinji Shimode, Tatsuki Toda

**Affiliations:** Soka University, Hachioji, Tokyo 192-8577, Japan; Atmosphere and Ocean Research Institute, The University of Tokyo, Kashiwa, Chiba 277-8564, Japan; Soka University, Hachioji, Tokyo 192-8577, Japan; Soka University, Hachioji, Tokyo 192-8577, Japan; National Institute for Environmental Studies, Tsukuba, Ibaraki 305-8506, Japan; National Institute for Environmental Studies, Tsukuba, Ibaraki 305-8506, Japan; Japan Agency for Marine-Earth Science and Technology, Yokosuka, Kanagawa 237-0061, Japan; Yokohama National University, Yokohama, Kanagawa 240-8501, Japan; Soka University, Hachioji, Tokyo 192-8577, Japan

**Keywords:** copepod nauplii, ingestion rate, food requirement, microzooplankton, microbial food web

## Abstract

To quantitatively evaluate the role of copepod nauplii as predators in the microbial food web, the ingestion rate (IR) of copepod nauplii and the food requirement (FR) of microzooplankton were estimated monthly for 3 consecutive years in temperate embayment waters. The IR of dominant copepod nauplii (*Acartia* spp. nauplii) was estimated from water temperature, individual carbon weight and food concentration and peaked (>0.50 μgC ind^−1^ d^−1^) with relatively high food concentration (>57.5 μgC L^−1^). This result suggests that food concentration should be considered to estimate copepod naupliar IR in marine environments, especially where biological conditions fluctuate largely. The comparison of copepod naupliar and microprotozoan FR showed the dominance of naked ciliate FR (77.0–90.2%) during the study period except in spring when comparable values were observed between the FR of naked ciliates (41.6%) and copepod nauplii (33.6%). During spring, transfer efficiency (10.5%) from primary production (PP) to microzooplankton production was lower than in other seasons (16.2–17.1%). This study indicates that copepod nauplii are seasonally important micro-sized predators in the microbial food web of temperate embayment waters and that carbon flow through copepod nauplii is a pathway which inefficiently transfers PP to higher trophic levels.

## INTRODUCTION

In marine pelagic waters, microbial communities transfer carbon and energy to higher trophic levels via predator–prey interactions through the microbial food web (Azam, 1998; Azam *et al.,* 1983). The microbial food web is comprised of various taxonomic groups of microorganisms ranging from pico-sized (0.2–2 μm) to micro-sized (20–200 μm) plankton including phytoplankton, heterotrophic bacteria, protozoans such as heterotrophic nanoflagellates (HNFs) and ciliates, and metazoans. The understanding of complex trophic interactions among the components of the microbial food web is essential to clarify the structure and function of marine ecosystems (Vargas *et al.,* 2007). Thus, the quantitative studies of carbon flow in the microbial food web and its temporal and spatial variations are required.

Among marine planktonic communities, the most abundant metazoans are copepod nauplii (López *et al.,* 2007a; White and Roman, 1992), the larvae of copepods in the first developmental stage (until the completion of the sixth molt). Copepod nauplii are widely distributed from equatorial to polar regions and from surface to deep layers (e.g. Dagg *et al.,* 1984; Lewis *et al.,* 1996). Based on their size, copepod nauplii are classified into microzooplankton and mainly feed on nano-sized (2–20 μm) plankton too small to be utilized efficiently by their adults (Berggreen *et al.,* 1988; Helenius and Saiz, 2017). Previous studies reported that copepod nauplii ingest more amount of food than their body weight within a day, which results in considerably higher feeding rate per body weight compared with their adults (Henriksen *et al.,* 2007; Lonsdale *et al.,* 1996; Paffenhöfer, 1971; Smith *et al.,* 2008). For example, daily carbon uptake by *Oithona* spp. nauplii in Coliumo Bay, Chile, reached up to 1107% of their body carbon (Böttjer *et al.,* 2010). Because copepod nauplii are the predators of nanoplankton and also the prey of mesozooplankton and fish larvae (Conway *et al.,* 1998; Dalpadado *et al.,* 2000), they are considered a trophic link between the components of the microbial food web and higher trophic levels (Turner, 2004; Turner and Roff, 1993). However, the information on the feeding ecology of copepod nauplii has remained relatively limited compared with copepodites and adults.

The ingestion rate (IR) of copepod nauplii depends on factors such as water temperature (WT), prey quality (e.g. size and taxa) and quantity (concentration), and their own carbon weight (CW, Almeda *et al.,* 2010; Helenius and Saiz, 2017; Henriksen *et al.,* 2007; Saiz and Calbet, 2007). Traditionally, although the IR of zooplankton has been calculated from respiration rate using WT, CW and the constants of assimilation and gross growth efficiency (Ikeda, 1985; Ikeda and Motoda, 1978), the respiration-based estimation may contain considerable inaccuracy for copepod nauplii. Feeding experiments are commonly conducted to obtain copepod naupliar IR using food removal (Frost, 1972; Gifford, 1993) and gut fluorescence (López *et al.,* 2007b; Mackas and Bohrer, 1976) techniques. However, the concentrations of the cultured or natural prey used in feeding experiments are often different from the *in situ* prey concentrations, and copepod naupliar IR estimated by these methods as described above does not reflect the effect of food concentration. Since copepod naupliar IR varies at different food concentration, IR considering food concentration should be estimated, especially in coastal waters where biological conditions fluctuate largely on a temporal scale.

Natori and Toda (2018) conducted feeding experiments using the nauplii of an embayment copepod *Acartia steueri*  Smirnov, 1936 (Copepoda: Calanoida), a dominant species in the inner bay of temperate areas around Asia (Kang and Kang, 2005; Onoue *et al.,* 2006; Smirnov, 1936; Uye, 1981). To elucidate the effect of WT, CW and food concentration on *A*. *steueri* naupliar IR, various developmental stages (NIII–NVI) of the nauplii were fed with different concentrations of prey at various WTs. By using the relationships between CW and the feeding parameters of a functional response model, an empirical model was constructed to estimate the carbon-specific IR of *A*. *steueri* nauplii using individual CW and food concentration, which can be supplemented by temperature quotient (*Q*_10_). They suggested that the empirical model can be widely applied to other species of copepod nauplii and enables the estimation of copepod naupliar IR which reflects the effect of the *in situ* food concentration in field investigations.

Therefore, the present study estimated the IR of copepod nauplii using the empirical model proposed by Natori and Toda (2018). Then, carbon flow from phytoplankton to microzooplankton (copepod nauplii and microprotozoans) was described to quantitatively evaluate the trophodynamic role of copepod nauplii in the microbial food web. This study was carried out in temperate embayment waters with large seasonal and irregular variations in the concentrations of copepod naupliar prey.

## METHOD

### Study area and samplings

**Fig. 1:**
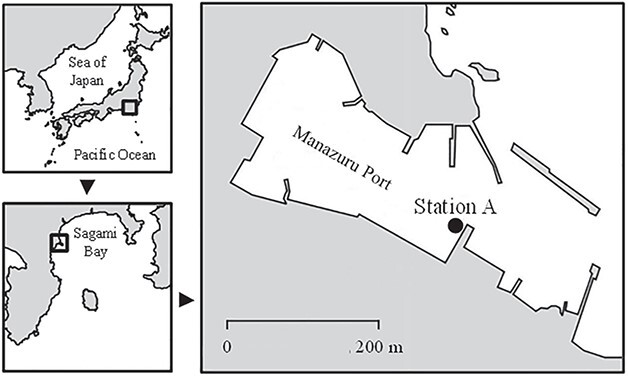
Location of Station A in Sagami Bay, Japan.

Monthly surveys were conducted for 3 years from November 2012 to November 2015 in Sagami Bay, Japan, located on the southern coast of central Japan and facing the northwestern Pacific Ocean. Sagami Bay is considered as one of the representative temperate coastal areas, and physical, chemical and biological environments have been intensively investigated for over 25 years in coastal waters of Sagami Bay (e.g. Ara *et al.,* 2011; Fujiki *et al.,* 2004; Kuwahara *et al.,* 2015; Sugai *et al.,* 2016). Samplings were carried out just after sunset at an embayment site, Station A (35°09′49″ N, 139°10′33″ E, maximum depth 6 m) where biological conditions change seasonally and abruptly due to the irregular inflow of freshwater and tidewater (Miyaguchi *et al.,* 2008; Onoue *et al.,* 2006; Satoh *et al.,* 2000; Tsuchiya *et al.,* 2013) (Fig. 1). The details of the study area and sampling station were described by Sugai *et al.* (2016) and Takayama and Toda (2019).

Seawater was collected at 1-m depth using a 5-L Niskin bottle. WT was measured immediately after the collection of seawater with a mercury thermometer. For the samples of salinity, chlorophyll *a* (chl. *a*), bacteria and HNFs, the collected seawater was transferred to the shaded polycarbonate bottle (5 L, Nalgene) after pre-filtering through 180-μm nylon mesh to remove large plankton and debris. For a microzooplankton sample, the collected seawater was transferred to the 10-L polycarbonate bottle without pre-filtration. Samples were brought back to a laboratory in the Manazuru Marine Center for Environmental Research and Education (Yokohama National University) within 15 minutes. For the sample of primary production (PP), the collected seawater was pre-filtered through 180-μm nylon mesh and transferred to 1-L polycarbonate bottles (Nalgene, three light bottles and one dark bottle). After the addition of ^13^C-NaHCO_3_ (final ^13^C atom % in total inorganic carbon < 10%, Cambridge Isotope Laboratories), the seawater was incubated for 24 h at the surface of Station A under the *in situ* WT and light (natural solar radiation) conditions (Hama *et al.,* 1983).

### Analytical methods

Salinity was measured with an inductive salinometer (601 Mk IV, Yeo-Kal Electronics). For the analysis of chl. *a*, seawater was size-fractionated using 2- and 10-μm pore size polycarbonate filters and 20-μm nylon mesh to obtain <2, 2–10, 2–20 and >20 μm chl. *a* concentration. Then, the seawater (200–400 mL) was filtered onto glass fiber filters (Whatman GF/F, GE Healthcare Life Sciences). Chl. *a* pigment on the filters was extracted with *N*,*N*-dimethylformamide and stored at −20°C until analysis (Suzuki and Ishimaru, 1990). Chl. *a* concentration was measured by a fluorometer (10-AU, Turner Designs) according to Welschmeyer (1994). Phytoplankton biomass was determined using a conversion factor of 40 μgC (μg Chl. *a*)^−1^ (Montagnes *et al.,* 1994; Parsons *et al.,* 1984). For the analysis of bacteria, seawater (10 mL) was fixed with the buffered and pre-filtered (<0.2 μm) formaldehyde (final concentration 2%) and preserved at −20°C until analysis. Bacterial cells were stained with a nucleic acid stain (SYBR Gold, Invitrogen) following Shibata *et al.* (2006) and filtered on 0.2-μm pore size polycarbonate filters (Nuclepore Track-Etch Membrane Black, GE Healthcare Life Sciences). On each filter, more than 400 cells were counted by examining at least 20 microscopic fields at ×1000 magnification using an epifluorescence microscope (Axioskop 2 plus, Zeiss). Bacterial biomass was calculated using a conversion factor of 34 fgC cell^−1^ (Hamasaki *et al.,* 1999; Kogure and Koike, 1987). For the analysis of HNFs, seawater (100 mL) was fixed with glutaraldehyde (final concentration 1%) and stored at 4°C until analysis. Protist cells were filtered on 0.8-μm pore size polycarbonate filters (Nuclepore Track-Etch Membrane Black, GE Healthcare Life Sciences) and stained with a protein stain (Primulin, Sigma-Aldrich) according to Sherr *et al.* (1993). On each filter, more than 50 cells were enumerated by examining at least 20 microscopic fields at ×1000 magnification using the epifluorescence microscope. HNFs were distinguished from autotrophic nanoflagellates by autofluorescent pigments. Cell size was measured with an eyepiece micrometer during counting, and cell volume was calculated based on geometric configuration. HNF biomass was estimated using a conversion factor of 220 fgC μm^−3^ (Børsheim and Bratbak, 1987).

For the analysis of microprotozoans, seawater (500 mL) was fixed with acid Lugol solution (final concentration 2%) and concentrated with an Utermöhl settling chamber (Hydro-Bios). Microprotozoans, namely naked ciliates, tintinnids and heterotrophic dinoflagellates (HDFs), were enumerated using an inverted microscope (DM IL LED, Leica) following Carey (1992) and Chihara and Murano (1997). Cell volume (CV, μm^3^) or lorica volume (LV, μm^3^) was calculated based on geometric configuration, and microprotozoan biomass (pgC) was estimated by 0.19 *CV* for naked ciliates (Putt and Stoecker, 1989), 0.053 *LV* + 444 for tintinnids (Verity and Langdon, 1984) and 0.284 *CV*  ^0.900^ for HDFs (Menden-Deuer and Lessard, 2000). For the analysis of copepod nauplii, seawater (10 L) was filtered through 20-μm nylon mesh and fixed with the buffered formaldehyde (final concentration 5%). Copepod nauplii were classified into *Acartia* spp., other Calanoida, Cyclopoida or Harpacticoida based on morphological characteristics (Dahms, 2006; Okada *et al.,* 2009) and counted using a dissecting microscope (WILD M10, Leica). Body length (BL, μm) was measured during counting, and individual CW (ngC ind^−1^) was determined by 6.46 × 10^−6^  *BL*  ^3.20^ for *Acartia* spp. nauplii (Natori and Toda, 2018) and 1.51 × 10^−5^  *BL*  ^2.94^ for other copepod nauplii (Uye *et al.,* 1996).

For the analysis of PP, the incubated seawater (200–400 mL) was filtered onto the pre-combusted (450°C, 4 h) glass fiber filters (Whatman GF/F, GE Healthcare Life Sciences). The concentration of particulate organic carbon and the isotope ratio of ^12^C and ^13^C were measured by an organic elemental analyzer (Flash 2000, Thermo Fisher Scientific) and a mass spectrometer facilitated with a combustion furnace (DELTA V Advantage, Thermo Fisher Scientific), respectively. With the assumption of the dissolved inorganic carbon concentration of 2.2 mM (Gao and McKinley, 1994), PP was calculated according to Hama *et al.* (1983).

### Calculations

The IR of *Acartia* spp., other calanoid and harpacticoid nauplii was determined by individual *CW* × carbon-specific *IR* (^s^I, d^−1^), which was calculated using the following empirical model:


$$ {}^{\mathrm{s}}I=1.07\times{10}^3\ {CW}^{-1.05}\ \frac{FC^2}{FC^2+{\left(1.35\ {CW}^{0.687}\right)}^2}, $$

where CW is carbon weight and FC is food concentration (μgC L^−1^) according to Natori and Toda (2018). The IR of cyclopoid nauplii (I^*^, cells ind^−1^ d^−1^) was calculated by


$$ {I}^{\ast }=8.87\ {CW}^{0.766}\ \frac{{FC^{\ast}}^2}{{FC^{\ast}}^2+{\left(259\ \left(1-{\mathrm{e}}^{-0.029\ CW}\right)\right)}^2}, $$

where FC^*^ is food concentration (cells mL^−1^) following Natori and Toda (2018). Based on the observation of the gut contents of *A. steueri* nauplii with a novel fracturing device and a scanning electron microscope by Natori *et al.* (2017), phytoplankton (2–10 μm) and HNFs were regarded as the *in situ* food items of copepod nauplii in this study. The effect of WT on ^s^I was supplemented by using *Q*_10_ (2.37) when WT was lower than 22°C and by multiplying ^s^I by 0.287 when WT was higher than 22°C (Natori and Toda, 2018). To correct I^*^, a *Q*_10_ of 2.45 was used (Almeda *et al.,* 2010).

The food requirement (FR) of microprotozoans was estimated by microprotozoan biomass × maximum specific *IR* (d^−1^), which was calculated using 30.2 *CV*  ^–0.20^ for naked ciliates, 30.2 *LV*  ^–0.20^ for tintinnids and 33.9 *CV*  ^–0.27^ for HDFs (Hansen *et al.,* 1997). The effect of WT on the microprotozoan IR was supplemented using a *Q*_10_ of 2.8 (Hansen *et al.,* 1997; Sherr *et al.,* 1988). The FR of copepod nauplii was determined by naupliar biomass × ^s^*I* for *Acartia* spp., other calanoid and harpacticoid nauplii and by naupliar abundance × I^*^ for cyclopoid nauplii. Microzooplankton production (MP) was estimated by microzooplankton biomass × specific growth rate (*G*, d^−1^), calculated using Ln *G* = − 0.27 Ln *CV* + 1.52 Ln *WT* − 1.44 for naked ciliates (Müller and Geller, 1993), Ln *G* = − 0.27 Ln *LV* + 1.52 Ln *WT* − 1.44 for tintinnids (Müller and Geller, 1993), 8.13 *CV*  ^–0.26^ and a *Q*_10_ of 2.8 for HDFs (Caron *et al.,* 1986; Caron *et al.,* 1990; Hansen *et al.,* 1997) and 0.057 e ^0.069 *WT*^ for copepod nauplii (Uye *et al.,* 1996), where WT is water temperature. The grazing impact of microzooplankton on phytoplankton was determined by *FR*/*PP*, and the gross growth efficiency of microzooplankton was estimated by *MP/FR*. Transfer efficiency from PP to MP was calculated by the ratio of MP to PP.

### Data analyses

Correlation analyses were performed using the Spearman’s rank correlation coefficients (*r_s_*), and probability less than 0.01 (*P* < 0.01) was considered significant. To describe the seasonality of the biomass of microbial communities, non-metric multidimensional scaling (nMDS) analysis was conducted using the Bray–Curtis similarity index. Microbial biomass was fitted to the nMDS ordination to examine the drivers of the distribution of monthly samples.

## RESULTS

### Environmental factors

**Fig. 2:**
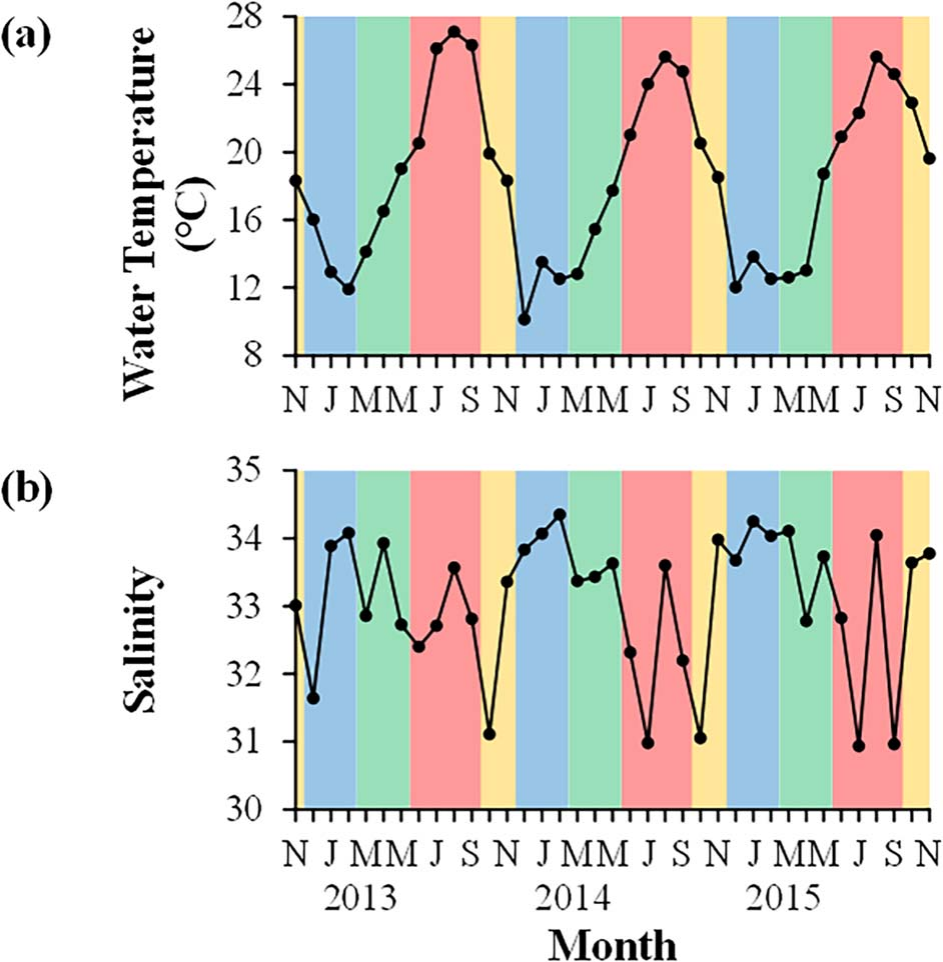
Monthly variations in (**a**) WT and (**b**) salinity at Station A from November 2012 to November 2015. Yellow, blue, green and red colors indicate autumn, winter, spring and summer, respectively (see Fig. 5).

WT ranged from 10.1°C in December 2013 to 27.1°C in August 2013 (Fig. 2a). WT showed clear seasonality every year: low during winter and high during summer. Salinity was relatively high (>31.6) during winter and spring and sometimes relatively low (<31.1) during autumn and summer (Fig. 2b). The highest (34.4) and lowest (30.9) values were observed in February 2014 and July 2015, respectively.

### Microbial biomass

**Fig. 3:**
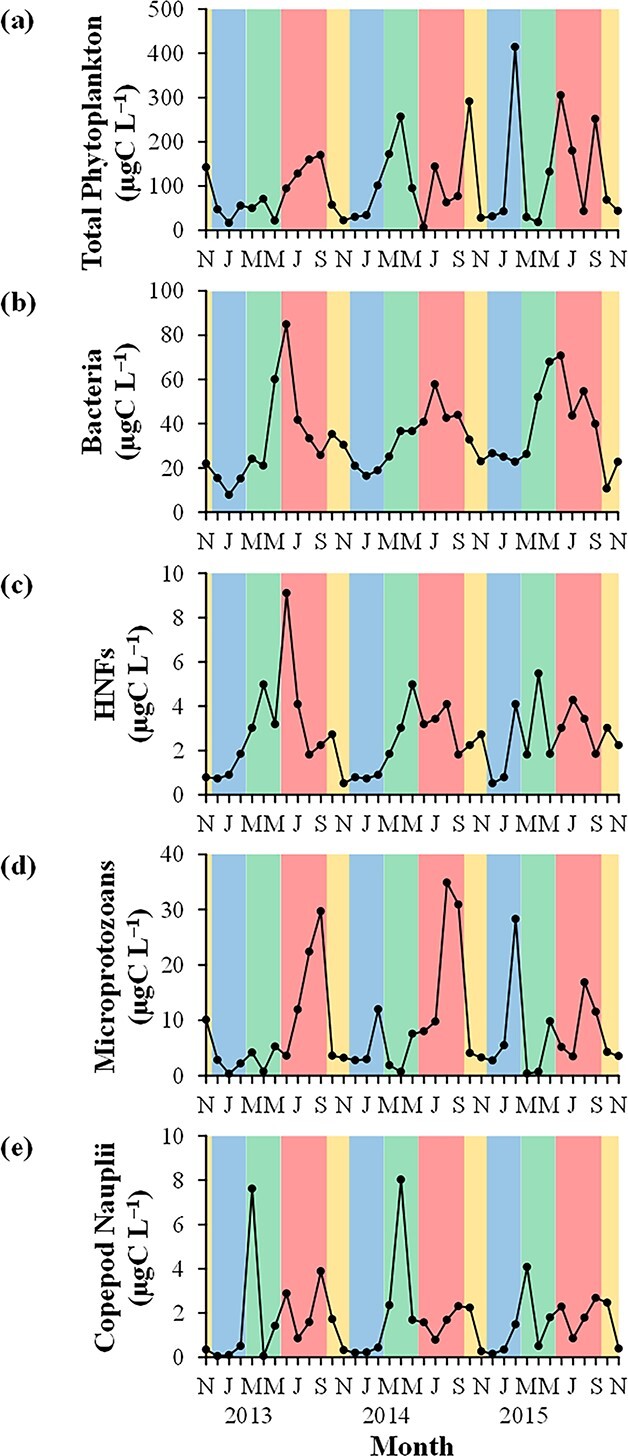
Monthly variations in the biomass of (**a**) total phytoplankton, (**b**) bacteria, (**c**) heterotrophic nanoflagellates (HNFs), (**d**) microprotozoans and (**e**) copepod nauplii at Station A from November 2012 to November 2015. Yellow, blue, green and red colors indicate autumn, winter, spring and summer, respectively (see Fig. 5).

**Fig. 4:**
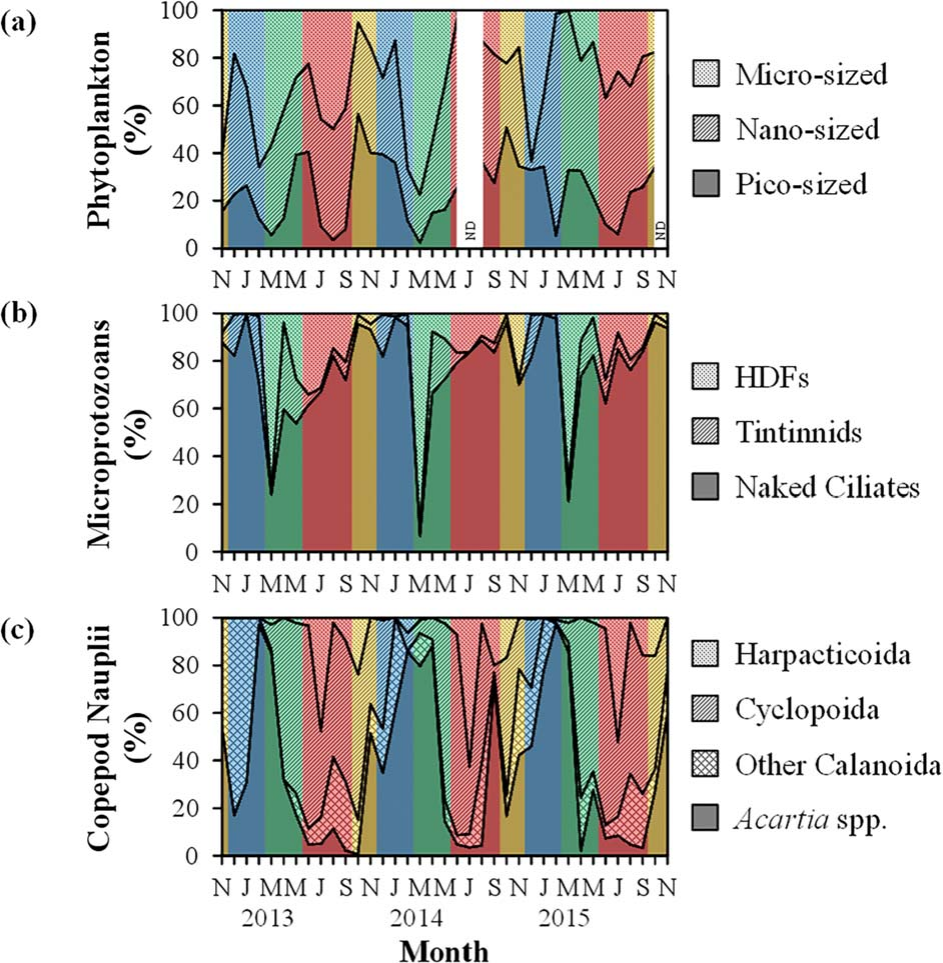
Monthly variations in the biomass compositions of (**a**) phytoplankton, (**b**) microprotozoans and (**c**) copepod nauplii at Station A from November 2012 to November 2015. Yellow, blue, green and red colors indicate autumn, winter, spring and summer, respectively (see Fig. 5). ND: no data.

The peaks of total phytoplankton biomass (>252 μgC L^−1^) occurred during all seasons, and clear seasonality was not observed (Fig. 3a). During the study period, picophytoplankton, nanophytoplankton and microphytoplankton accounted for 24.2 ± 14.2% (mean ± SD), 44.5 ± 17.4% and 32.6 ± 21.1% of total phytoplankton, respectively (Fig. 4a). The proportion of nanophytoplankton biomass was the highest in February 2015 (93.2%) but sometimes relatively high (>65.1%) during summer and spring. Bacterial biomass ranged from 7.92 μgC L^−1^ in January 2013 to 84.9 μgC L^−1^ in June 2013 (Fig. 3b), and HNF biomass varied from 0.52 μgC L^−1^ in November 2013 and December 2014 to 9.11 μgC L^−1^ in June 2013 (Fig. 3c). Generally, bacterial and HNF biomass showed high values during spring and summer and low values during autumn and winter.

Microprotozoan biomass was often relatively high (>22.4 μgC L^−1^) during summer and in February 2015 (28.3 μgC L^−1^) (Fig. 3d). The lowest value was observed in January 2013 (0.33 μgC L^−1^). Among microprotozoans, naked ciliates were dominant (76.3 ± 21.7%) during the study period, whereas tintinnids and HDFs comprised 8.00 ± 8.94% and 15.7 ± 21.9% of microprotozoans, respectively (Fig. 4b). The proportion of naked ciliate biomass often showed relatively low values (<23.9%) during spring. The biomass of copepod nauplii peaked (>7.62 μgC L^−1^) during spring (Fig. 3e). During the study period, *Acartia* spp. nauplii (36.9 ± 32.9%) and cyclopoid nauplii (36.8 ± 29.5%) dominated copepod nauplii while other calanoid nauplii and harpacticoid nauplii accounted for 18.0 ± 18.6% and 8.3 ± 15.3% of copepod nauplii, respectively (Fig. 4c). The proportion of *Acartia* spp. naupliar biomass was often relatively high (>85.1%) during winter and spring.

### Relationships between parameters

WT was significantly positively correlated with the biomass of bacteria (*r_s_* = 0.548, *n* = 37, *P* < 0.001), naked ciliates (*r_s_* = 0.603, *n* = 37, *P* < 0.001) and HDFs (*r_s_* = 0.670, *n* = 37, *P* < 0.001) during the study period (Table I). Significant positive correlations were also observed between the biomass of nanophytoplankton and naked ciliates (*r_s_* = 0.436, *n* = 35, *P* < 0.01) or copepod nauplii (*r_s_* = 0.531, *n* = 35, *P* < 0.01).

**Table I:** Spearman’s rank correlation coefficients among environmental factors and the biomass of microbial communities during the study period

Parameter	Bac	HNF	NC	Tin	HDF	CN
WT	0.548^a^	0.364	0.603^a^	0.258	0.670^a^	0.391
Sal	–0.525^a^	-0.277	-0.139	-0.015	-0.335	-0.293
PicoP	0.251	0.086	0.397	0.172	-0.079	-0.366
NanoP	0.288	0.300	0.436^b^	0.326	0.345	0.531^b^
MicroP	0.087	0.044	0.158	0.190	0.283	0.271
Bac		0.506^b^	0.246	0.263	0.575^a^	0.451^b^
HNF			0.058	0.126	0.359	0.339
NC				0.522^a^	0.401	0.156
Tin					0.275	0.143
HDF						0.526^a^
CN						

Sal: salinity; PicoP: picophytoplankton; NanoP: nanophytoplankton; MicroP: microphytoplankton; Bac: bacteria; HNF: heterotrophic nanoflagellate; NC: naked ciliate; Tin: tintinnid; HDF: heterotrophic dinoflagellate; CN: copepod nauplius.

^a^Significant at *P* < 0.001.

^b^Significant at *P* < 0.01

### Seasonality of microbial biomass

**Fig. 5:**
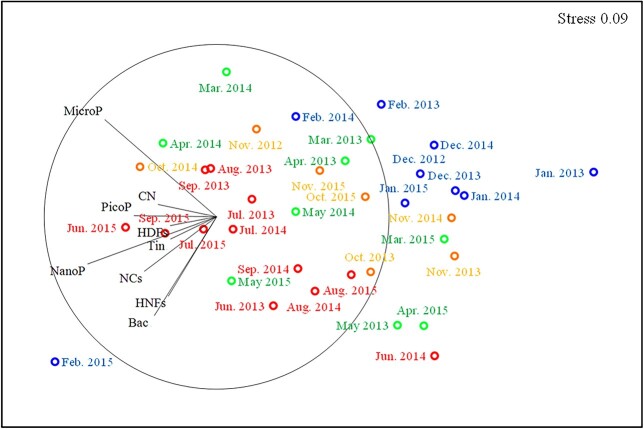
Non-metric multidimensional scaling analysis showing the distribution of monthly samples at Station A from November 2012 to November 2015 and the vectors of the biomass of microbial communities. Yellow, blue, green and red circles represent autumn, winter, spring and summer samples, respectively (see text). PicoP: picophytoplankton; NanoP: nanophytoplankton; MicroP: microphytoplankton; Bac: bacteria; HNFs: heterotrophic nanoflagellates; NCs: naked ciliates; Tin: tintinnids; HDFs: heterotrophic dinoflagellates; CN: copepod nauplii.

nMDS analysis showed the seasonal change of the biomass of microbial communities (Fig. 5). Based on the distribution, monthly samples were grouped into autumn (October–November), winter (December–February), spring (March–May) and summer (June–September). Generally, summer season was characterized by high microbial biomass, whereas low microbial biomass was observed during winter except in February 2015 when the biomass of nanophytoplankton (387 μgC L^−1^) and naked ciliates (27.7 μgC L^−1^) was relatively high. Autumn and spring samples were distributed between summer and winter samples and showed the similar composition of microbial communities.

### IR and FR

**Fig. 6:**
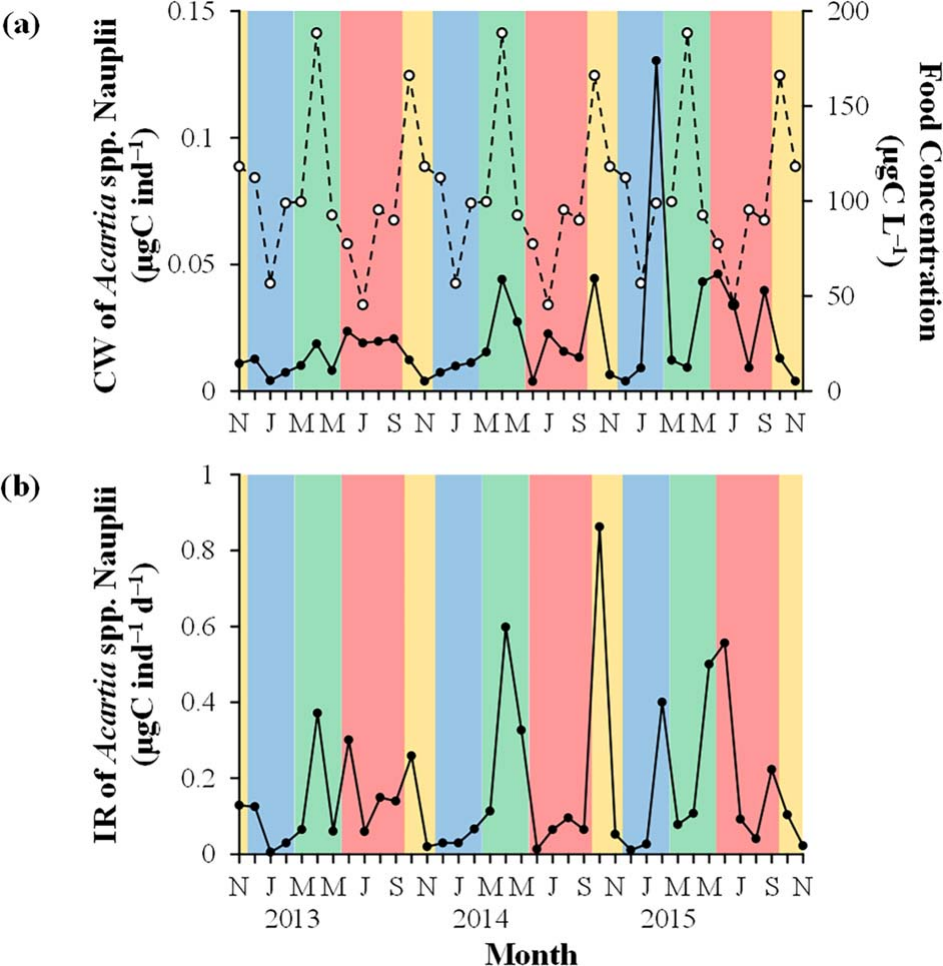
Monthly variations in (**a**) the individual CW of *Acartia* spp. nauplii (open circles) and their food concentration (closed circles) and (**b**) the IR of *Acartia* spp. nauplii at Station A from November 2012 to November 2015. Yellow, blue, green and red colors indicate autumn, winter, spring and summer, respectively (see Fig. 5).

The individual CW of *Acartia* spp. nauplii generally showed relatively high values during autumn and spring and relatively low values during winter and summer (Fig. 6a). Their food concentration was the lowest in June 2014 (5.09 μgC L^−1^), and the peaks (>52.9 μgC L^−1^) were observed during all seasons without clear seasonality. The IR of *Acartia* spp. nauplii varied from 0.01 μgC ind^−1^ d^−1^ in January 2013 to 0.86 μgC ind^−1^ d^−1^ in October 2014 (Fig. 6b). *Acartia* spp. naupliar IR peaked (>0.50 μgC ind^−1^ d^−1^) during spring, autumn and summer.

**Fig. 7:**
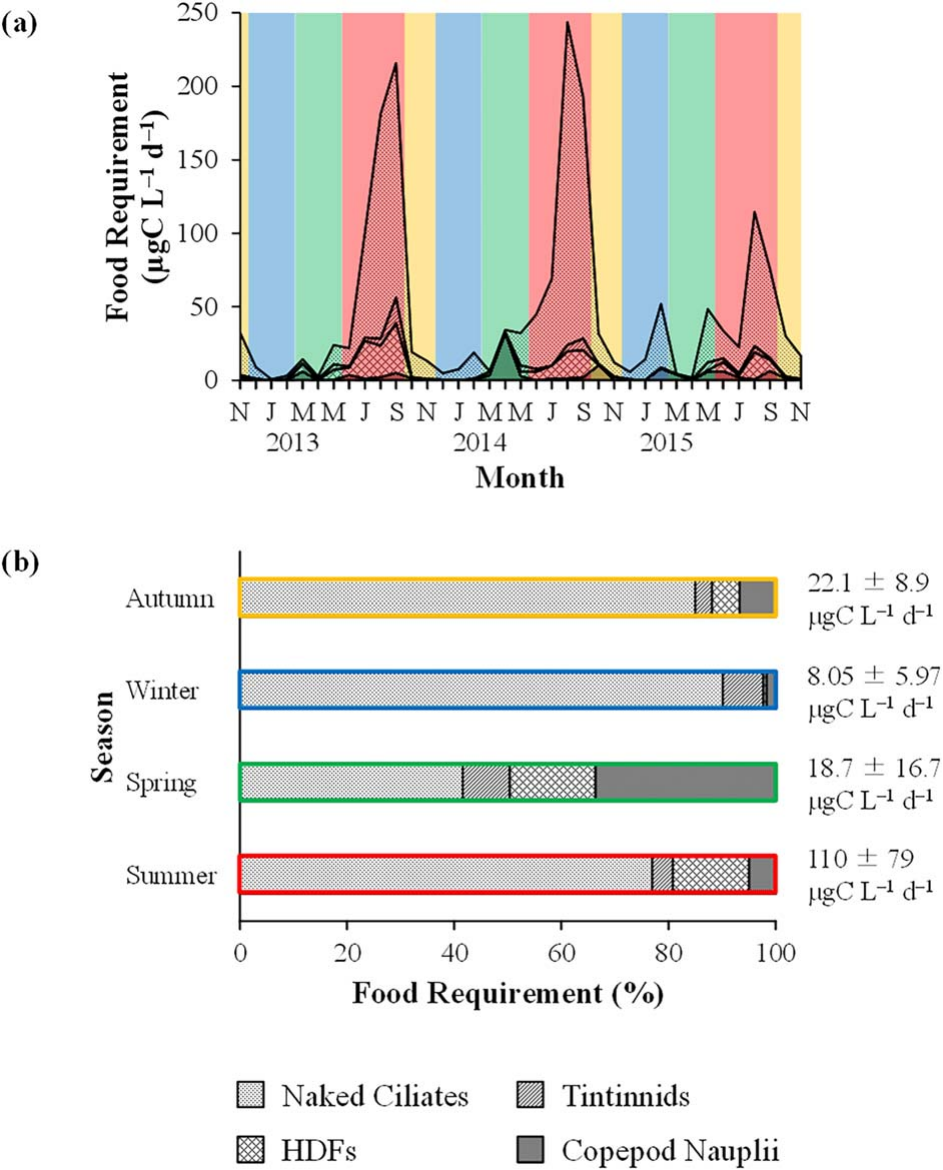
(**a**) Monthly and (**b**) seasonal variations in the FR of microzooplankton at Station A from November 2012 to November 2015. For (**b**), February 2015 was excluded from winter samples, and microzooplankton FR during each season is also shown. Yellow, blue, green and red colors indicate autumn, winter, spring and summer, respectively (see Fig. 5).

The FR of microprotozoans ranged from 0.21 μgC L^−1^ d^−1^ (March 2015) to 220 μgC L^−1^ d^−1^ (August 2014) for naked ciliates, from 0 μgC L^−1^ d^−1^ (January 2013 and July 2014) to 17.2 μgC L^−1^ d^−1^ (September 2013) for tintinnids and from 0 μgC L^−1^ d^−1^ (January 2013) to 33.9 μgC L^−1^ d^−1^ (September 2013) for HDFs (Fig. 7a). Naked ciliate FR was often relatively high (>153 μgC L^−1^ d^−1^) during summer. The FR of copepod nauplii varied from 0.01 μgC L^−1^ d^−1^ in January 2013 to 31.9 μgC L^−1^ d^−1^ in April 2014. Relatively high copepod naupliar FR (>8.04 μgC L^−1^ d^−1^) was sometimes observed during spring, autumn and winter. Seasonally, the FR of microzooplankton showed a much higher value during summer (110 ± 79 μgC L^−1^ d^−1^) compared with other seasons (<22.1 ± 8.9 μgC L^−1^ d^−1^) (Fig. 7b). Microzooplankton FR was dominated by naked ciliates during autumn (85.0 ± 10.4%), winter (90.2 ± 9.6%) and summer (77.0 ± 11.1%). During spring, the proportion of the FR of naked ciliates and copepod nauplii varied widely (4.67–74.6% and 1.85–93.0%, respectively) and showed comparable values (41.6 ± 31.9% and 33.6 ± 36.2%, respectively).

### Carbon flow from phytoplankton to microzooplankton

**Fig. 8:**
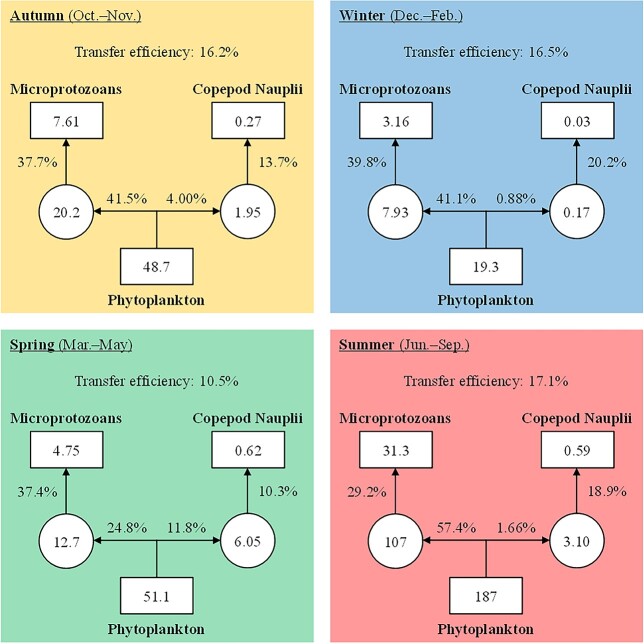
Seasonality of carbon flow from phytoplankton to microzooplankton at Station A from November 2012 to November 2015 except February 2015. Numbers in squares and circles are production and FR (μgC L^−1^ d^−1^), respectively. Percentages are the ratios of production and FR and mean the feeding impact or assimilation efficiency of microzooplankton. Transfer efficiency from PP to MP is also shown.

PP, microprotozoan production and copepod naupliar production were the lowest during winter (19.3 ± 10.6 μgC L^−1^ d^−1^, 3.16 ± 2.28 μgC L^−1^ d^−1^ and 0.03 ± 0.02 μgC L^−1^ d^−1^, respectively), and the highest values were observed during summer for PP (187 ± 74 μgC L^−1^ d^−1^) and microprotozoan production (31.3 ± 21.9 μgC L^−1^ d^−1^) and during spring for copepod naupliar production (0.62 ± 0.43 μgC L^−1^ d^−1^) (Fig. 8). The grazing impact of microprotozoans on phytoplankton showed a relatively high value during summer (57.4%) and low value during spring (24.8%), while copepod naupliar grazing impact was relatively high during spring (11.8%). The gross growth efficiency of copepod nauplii ranged from 10.3% during spring to 20.2% during winter and showed lower values compared with that of microprotozoans (29.2–39.8%) during all seasons. Lower transfer efficiency from PP to MP was observed during spring (10.5%) relative to other seasons (16.2–17.1%).

## DISCUSSION

### Seasonality of microbial biomass

During the study period, total phytoplankton biomass did not show clear seasonality (Fig. 3a). Relatively large phytoplankton blooms occurred during spring (April 2014), autumn (October 2014) and late-winter (February 2015) as typically observed in temperate coastal areas (e.g. Carstensen *et al.,* 2015; Cebrián and Valiela, 1999) and also during summer (June 2015 and September 2015). This is consistent with previous studies which conducted the weekly based field surveys at the same study site (Station A). They reported the abrupt increase in phytoplankton abundance during summer due to complex water masses formed by freshwater and tidewater inflow (Onoue *et al.,* 2006; Satoh *et al.,* 2000). The present study confirmed both the seasonal and irregular variations in phytoplankton biomass at an embayment station.

The biomass of bacteria, HNFs and microprotozoans was relatively high during summer and low during winter except in February 2015 (Fig. 3b–d). In addition, WT was significantly positively correlated with the biomass of some heterotrophic microbial communities, and nanophytoplankton biomass showed a significant positive correlation with naked ciliate biomass during the study period (Table I). These results generally indicate the bottom-up control of those heterotrophic microbial communities due to the dependency of their biomass on WT and food availability as reported by previous studies (Ara and Hiromi, 2009; Dupuy *et al.,* 2011). On the other hand, copepod naupliar biomass showed peaks during spring (March 2013 and April 2014) when *Acartia* spp. nauplii dominated (Figs 3e and 4c), and no relationship was observed between WT and copepod naupliar biomass (Table I). These may be partly because high WT is not appropriate for *A. steueri* (Natori and Toda, 2018; Uye, 1981), which were dominant at Station A most of the year except in summer and the most abundant during spring (Onoue *et al.,* 2006).

### IR and FR

In this study, an empirical model (Natori and Toda, 2018) was used to estimate the IR of *Acartia* spp. nauplii in natural environments. To construct the empirical model, Natori and Toda (2018) conducted feeding experiments using the nauplii (133–263 μm) and a haptophyte *Isochrysis galbana* (4.5 μm) according to the optimum predator/prey size ratio (1.71–3.38%) determined by the observation of the gut contents of the nauplii collected at Station A (Natori *et al.,* 2017). In the present study, *Acartia* spp. naupliar IR was calculated on the assumption that phytoplankton (2–10 μm) and HNFs were their food items in the *in situ* environments based on the observation by Natori *et al.* (2017). Thus, in terms of the size of their prey used in the feeding experiments (Natori and Toda, 2018) and assumed as food in the present study, the empirical model is relatively applicable to the estimation of the IR of *Acartia* spp. nauplii in this study. However, the effect of factors such as grazing on other food items (e.g. detritus particles) in natural environments, the taxa and motility of prey, and selective feeding on multiple prey should also be considered in addition to prey size (Helenius and Saiz, 2017; Henriksen *et al.,* 2007). Nevertheless, the empirical model enables the estimation of copepod naupliar IR, which reflects the effect of food concentration using the *in situ* biological parameters. The development of a better empirical model requires further studies.

The mean of *Acartia* spp. naupliar IR during the study period (0.17 μgC ind^−1^ d^−1^) was comparable to the mean IR of the nauplii of *A. hudsonica* (0.13 μgC ind^−1^ d^−1^) and *Acartia tonsa* (0.28 μgC ind^−1^ d^−1^) during spring–autumn in Chesapeake Bay (White and Roman, 1992) and *A. tonsa* during summer at Stony Brook Harbor (0.23 μgC ind^−1^ d^−1^) (Smith *et al.,* 2008). However, relatively high values of the *Acartia* spp. naupliar IR of the present study (>0.50 μgC ind^−1^ d^−1^), which were observed under the condition of relatively high food concentration (>57.5 μgC L^−1^) (Fig. 6a and b), were higher than the maximum IR of *A. tonsa* nauplii (0.32 μgC ind^−1^ d^−1^) at Stony Brook Harbor (Smith *et al.,* 2008).

To examine the relative importance of factors affecting *Acartia* spp. naupliar IR, the standard partial regression coefficients of WT, CW and food concentration were compared by performing a multiple regression analysis during the study period using the IR as a dependent variable (*R*^2^ = 0.530, *n* = 37, *P* < 0.001, ^*^: significant at *P* < 0.01, ^**^: significant at *P* < 0.001):


$$ IR=0.146\ WT+0.423\ {CW}^{\ast }+0.619\ {FC}^{\ast \ast }. $$

This analysis showed that CW and food concentration were significantly important factors and that food concentration exerted a larger effect on the IR compared with CW. Indeed, the peaks of the IR in April 2014, October 2014, May 2015 and June 2015 coincided with those of food concentration rather than CW (Fig. 6a and b). These results suggest that food concentration should be considered to estimate the IR of copepod nauplii in marine environments, especially in embayment and coastal waters where biological conditions fluctuate largely. Despite the peaks of food concentration, *Acartia* spp. naupliar IR was not very high in February 2015 and September 2015 due to low (12.5°C) and too high (24.6°C) WT for *Acartia* spp. nauplii (Fig. 2a).

The FR of microzooplankton during the study period (0.78–244 μgC L^−1^ d^−1^) (Fig. 7a) showed similar values with that in the neritic area of Sagami Bay throughout the year (2.8–273 μgC L^−1^ d^−1^) (Ara and Hiromi, 2009). Naked ciliates dominated microzooplankton FR during autumn, winter and summer (Fig. 7b). During spring, on the other hand, relatively low (8.86 ± 12.61 μgC L^−1^ d^−1^) and high (6.05 ± 9.97 μgC L^−1^ d^−1^) FR was observed for naked ciliates and copepod nauplii, respectively. As a result, the FR of copepod nauplii accounted for 33.6 ± 36.2% (1.85–93.0%) of microzooplankton FR and often exceeded naked ciliate FR during spring. Quevedo and Anadón (2000) found that copepod naupliar FR (20.3%) was the second largest among microzooplankton FR in the Bay of Biscay during spring and suggested the importance of copepod nauplii in microzooplankton communities in terms of grazing. These results indicate that, although naked ciliates are dominant on an annual basis, copepod nauplii are seasonally important micro-sized predators in the microbial food web of temperate embayment waters.

### Carbon flow from phytoplankton to microzooplankton

The grazing impact of microzooplankton on phytoplankton was 45.5, 42.0 and 59.1% during autumn, winter and summer, respectively (Fig. 8), which are comparable to the mean grazing impact observed in the coastal Mediterranean Sea off Barcelona (55.8%) (Calbet *et al.,* 2008) and in the neritic area of Sagami Bay (49.2%) (Ara and Hiromi, 2009) throughout the year. Microprotozoan grazing impact (41.1–57.4%) showed much higher values compared with copepod naupliar grazing impact (0.88–4.00%) during the seasons. This result agrees with Calbet *et al.* (2008) who found that microprotozoans were mainly responsible for grazing on phytoplankton because of their high biomass and specific IR. In contrast, during spring, the grazing impact of microprotozoans (24.8%) and copepod nauplii (11.8%) at a ratio of about 2:1 was observed mainly due to relatively low biomass of microprotozoans (3.47 ± 3.46 μgC L^−1^) and relatively high biomass of copepod nauplii (3.06 ± 2.93 μgC L^−1^) (Fig. 3d and e). Copepod naupliar grazing impact during spring was similar with the mean value (12.1%) reported during spring–autumn in Chesapeake Bay (White and Roman, 1992).

The gross growth efficiency of copepod nauplii (10.3–20.2%) was lower than that of microprotozoans (29.2–39.8%) during all seasons (Fig. 8). Microprotozoan gross growth efficiency of 40% is often assumed (Caron and Goldmann, 1990; Fenchel, 1987), and Almeda *et al.* (2010) reported the comparable gross growth efficiency of *Oithona davisae* nauplii (NII-NIII) (16–24%). These results indicate that more amount of carbon required by copepod nauplii is not used for their growth compared with microprotozoans. This is probably because copepod nauplii are multicellular organisms and have higher respiratory carbon loss relative to unicellular microprotozoans. Furthermore, copepod nauplii may have lost their food due to sloppy feeding as observed for the nauplii of *Paracartia grani* (Helenius and Saiz, 2017) and the undeveloped gut (Böttjer *et al.,* 2010).

In the simplified food web structure (Fig. 8), most of the carbon fixed by phytoplankton flowed through microprotozoans during autumn, winter and summer, which resulted in the transfer efficiency of 16.2–17.1%. On the other hand, during spring, PP was consumed by microprotozoans and copepod nauplii at a ratio of approximately 2:1, and lower transfer efficiency (10.5%) was observed. This is mainly due to higher FR and low gross growth efficiency of copepod nauplii. These results suggest that carbon flow through copepod nauplii increases seasonally (during spring) and transfers PP to higher trophic levels less efficiently compared with microprotozoans. However, carbon lost by their sloppy feeding is released as dissolved organic carbon and may be used by heterotrophic bacteria as an important carbon source (Møller, 2007).

## CONCLUSIONS

This study showed the temporal variation in copepod naupliar IR and compared the FR of copepod nauplii and microprotozoans in order to quantitatively assess their importance as predators in the microbial food web of temperate embayment waters. The IR of copepod nauplii, which was estimated using empirical models and the *in situ* biological parameters, largely fluctuated and clearly reflected the effect of the concentrations of their prey. Seasonally, copepod nauplii exerted a significant grazing pressure on the components of the microbial food web, but inefficient energy transfer from primary producers to higher trophic levels through copepod nauplii is suggested. Future carbon flow studies in the pelagic food web should incorporate the effect of prey quality and selective grazing into the estimation of copepod naupliar IR for better understanding of their *in situ* feeding rate and should investigate the fate of copepod nauplii in marine pelagic waters.

## Supplementary Material

JPR_Sugai_SupplementaryTable1_fbad002

## Data Availability

Raw data used in this study are available online in Soka University Repository (https://soka.repo.nii.ac.jp/, No. 32690 165).
