# Supplementary material for: Ingestion rate estimated from food concentration and predatory role of copepod nauplii in the microbial food web of temperate embayment waters
Source: J Plankton Res. 2023 Feb 1;45(2):325–37. doi: 10.1093/plankt/fbad002 (PMC10066810; doi:10.1093/plankt/fbad002)
Supplement: JPR_Sugai_SupplementaryTable1_fbad002 [file jpr_sugai_supplementarytable1_fbad002.docx]

**Supplementary Table I.** List of the meanings of abbreviations

| **Abbreviation** | **Meaning** |
| --- | --- |
| BL | Body length |
| Chl. *a* | Chlorophyll *a* |
| CV | Cell volume |
| CW | Carbon weight |
| FC | Food concentration (µgC L^–1^) |
| FC^*^ | Food concentration (cells mL^–1^) |
| FR | Food requirement |
| G | Specific growth rate |
| HDF | Heterotrophic dinoflagellate |
| HNF | Heterotrophic nanoflagellate |
| IR | Ingestion rate (µgC ind^–1^ d^–1^) |
| I^*^ | Ingestion rate (cells ind^–1^ d^–1^) |
| LV | Lorica volume |
| MP | Microzooplankton production |
| nMDS | Non-metric multidimensional scaling |
| PP | Primary production |
| *Q*_10_ | Temperature quotient |
| r_s_ | Spearman’s rank correlation coefficient |
| ^s^I | Carbon-specific ingestion rate (d^–1^) |
| WT | Water temperature |
